# Fast-Spiking Interneurons of the Premotor Cortex Contribute to Initiation and Execution of Spontaneous Actions

**DOI:** 10.1523/JNEUROSCI.0750-22.2023

**Published:** 2023-06-07

**Authors:** Nadia Giordano, Claudia Alia, Lorenzo Fruzzetti, Maria Pasquini, Giulia Palla, Alberto Mazzoni, Silvestro Micera, Leonardo Fogassi, Luca Bonini, Matteo Caleo

**Affiliations:** ^1^Neuroscience Institute, National Research Council (CNR), Pisa 56124, Italy; ^2^Scuola Normale Superiore, Pisa 56127, Italy; ^3^The BioRobotics Institute, Scuola Superiore Sant'Anna, Pontedera 56025, Italy; ^4^Department of Excellence in Robotics & IA, Scuola Superiore Sant'Anna, Pisa 56127, Italy; ^5^Centre for Neuroprosthetics and Institute of Bioengineering, Bertarelli Foundation Chair in Translational NeuroEngineering Laboratory, École Polytechnique Fédérale de Lausanne (EPFL), Lausanne 1015, Switzerland; ^6^Department of Medicine and Surgery, University of Parma, Parma 43125, Italy; ^7^Department of Biomedical Sciences, University of Padua, Padua 35121, Italy

**Keywords:** electrophysiology, fast spiking neurons, licking, premotor cortex, pyramidal neurons, single unit activity

## Abstract

Planning and execution of voluntary movement depend on the contribution of distinct classes of neurons in primary motor and premotor areas. However, timing and pattern of activation of GABAergic cells during specific motor behaviors remain only partly understood. Here, we directly compared the response properties of putative pyramidal neurons (PNs) and GABAergic fast-spiking neurons (FSNs) during spontaneous licking and forelimb movements in male mice. Recordings centered on the face/mouth motor field of the anterolateral motor cortex (ALM) revealed that FSNs fire longer than PNs and earlier for licking, but not for forelimb movements. Computational analysis revealed that FSNs carry vastly more information than PNs about the onset of movement. While PNs differently modulate their discharge during distinct motor acts, most FSNs respond with a stereotyped increase in firing rate. Accordingly, the informational redundancy was greater among FSNs than PNs. Finally, optogenetic silencing of a subset of FSNs reduced spontaneous licking movement. These data suggest that a global rise of inhibition contributes to the initiation and execution of spontaneous motor actions.

**SIGNIFICANCE STATEMENT** Our study contributes to clarifying the causal role of fast-spiking neurons (FSNs) in driving initiation and execution of specific, spontaneous movements. Within the face/mouth motor field of mice premotor cortex, FSNs fire before pyramidal neurons (PNs) with a specific activation pattern: they reach their peak of activity earlier than PNs during the initiation of licking, but not of forelimb, movements; duration of FSNs activity is also greater and exhibits less selectivity for the movement type, as compared with that of PNs. Accordingly, FSNs appear to carry more redundant information than PNs. Optogenetic silencing of FSNs reduced spontaneous licking movement, suggesting that FSNs contribute to the initiation and execution of specific spontaneous movements, possibly by sculpting response selectivity of nearby PNs.

## Introduction

Activity occurring before the initiation of voluntary movements is critical for action planning and execution (Weinrich and Wise, 1982; Godschalk et al., 1985; Wise and Mauritz, 1985; Churchland et al., 2006; Guo et al., 2014; Murakami et al., 2014; Li et al., 2016; Gao et al., 2018). Specifically, premotor areas act as a conductor to orchestrate the network activity of the rest of the motor modules, on a moment-by-moment basis, and exhibit tuning for specific movements (Georgopoulos et al., 1982; Godschalk et al., 1985; Hocherman and Wise, 1991; Riehle and Requin, 1993; Messier and Kalaska, 2000; Churchland et al., 2006; Churchland and Shenoy, 2007). How do distinct neuronal classes contribute to this process? The anticipatory activity of pyramidal neurons (PNs) has been previously examined (Svoboda and Li, 2018), however little is known on the contribution of GABAergic cells to these cortical computations (Merchant et al., 2008; Isomura et al., 2009; Kaufman et al., 2013).

Fast-spiking neurons (FSNs) are the most prevalent type of GABAergic interneurons in the cortex (Lourenço et al., 2020a) and are well suited to shape neuronal dynamics (Merchant et al., 2008; Isomura et al., 2009; Pi et al., 2013; Polack et al., 2013; Sachidhanandam et al., 2016). FSNs exhibit narrow action potentials and high spontaneous discharge rates (Merchant et al., 2012; Swanson and Maffei, 2019). In the rodent sensory cortex, FSNs contribute to sharpening the tuning of cortical neurons to preferred stimuli (Wu et al., 2008; Poo and Isaacson, 2009; Isaacson and Scanziani, 2011; Liu et al., 2011; Tan et al., 2011). In mice primary motor cortex, they fire before PNs during reaching movements (Estebanez et al., 2017), supporting a dynamic role of inhibition in shaping the tuning of PNs while routing information to the subsequent motor module (Merchant et al., 2008).

Here, we recorded premotor neuronal activity from the anterolateral motor cortex (ALM; Komiyama et al., 2010; Guo et al., 2014; Chen et al., 2017), which partially overlaps with the rostral forelimb area (RFA; Tennant et al., 2011; Vallone et al., 2016), in head-restrained mice allowed to either spontaneously lick or pull a handle in a robotic device (Spalletti et al., 2017). We found that FSNs fire longer than PNs and earlier during licking, but not forelimb movements. PNs displayed more specific activity during movement performed with different effectors (i.e., licking and arm retraction), while most FSNs increased their discharge regardless of the movement type. Computational analyses revealed that FSNs carried a greater amount of redundant information before PNs activation. Finally, optogenetic silencing of FSNs reduced spontaneous licking movement, suggesting that a global rise of inhibition contributes to the initiation and execution of spontaneous motor actions.

## Materials and Methods

### Experimental design and subject details

All experiments were conducted in accordance with the EU Council Directive 2010/63/EU and the Italian decree 26/2014 on the protection of animals used for scientific purposes and were approved by the Italian Ministry of Health (authorization number 753/2015-PR). Animals were housed in rooms at 22°C with a standard 12/12 h light/dark cycle. Food (standard diet, 4RF25 GLP Certificate, Mucedola) and water were available *ad libitum*, except for the experimental period, during which mice were water-deprived overnight. Electrophysiological recordings were conducted on 13 subjects. Experiments were conducted on three- to five-month-old wild-type (C57BL6/J) male mice. Six B6.Cg-Tg (Thy1-COP4/EYFP)18Gfng (ChR2) male mice expressing channelrhodopsin-2 (ChR2) mainly in corticofugal, Layer V neurons were used to map mouth/tongue movements in the ALM. For optotagging of FSNs, two PV-Cre male mice (Tanahira et al., 2009; B6;129P2-Pvalb tm1 (cre)Arbr/J, The Jackson Laboratory) were injected with an excitatory ChR2-expressing AAV vector (see details below). Finally, for inhibition of FSNs during licking activity, four PV-Cre mice were injected with a modified inhibitory ChR2-expressing AAV vector (see details below).

### Surgery procedure and animal preparation

#### Viral injections

PV-Cre mice were deeply anesthetized with an intraperitoneal injection of avertin (0.020 ml/g) and positioned on a stereotaxic frame; cranial sutures were exposed and used as reference. A small craniotomy was performed on the right ALM (1.8 mm lateral and 2.5 mm anterior to bregma). For optotagging the FSNs, we injected 600 nl of the AAV9/2 vector (pssAAV-2-hEF1α-dlox-hChR2(H134R)_mCherry(rev)-dlox-WPRE-hGHp(A), ETH Zurich Viral Vector Facility, 5.4 × 10^12^ vg/ml) containing the double floxed ChR2 fused to an mCherry reporter, thus expressed selectively in parvalbumin (PV) interneurons through Cre-mediated recombination (Spalletti et al., 2017; Tantillo et al., 2020), henceforth referred to as PV+ FSNs. For PV+ FSNs optogenetic inhibition experiments, we injected 600 nl of the AAV1 vector (pAAV_hSyn1-SIO-stGtACR2-FusionRed, 105677-AAV1, Addgene, 1.8 × 10^13^ vg/ml) containing the double floxed modified soma-targeted anion-conducting ChR2 fused to FusionRed, allowing its selective expression in PV+ FSNs. Viral vectors were injected using a microinjector (Nanoliter 2020 Injector, WPI), with an infusion rate of 90 nl/min at a 750-µm depth from the cortical surface. Skin was sutured and animals allowed to awaken. Three weeks later, injected mice underwent surgical procedure for electrophysiological or behavioral experiment.

#### Surgical preparation for electrophysiological and behavioral experiments

Mice were deeply anesthetized and positioned on a stereotaxic frame; the scalp was partially removed, the skull cleaned and dried. A custom-made lightweight headpost, was placed on the skull on the left hemisphere, aligned with the sagittal suture and cemented in place with a dental adhesive system (Super-Bond C&B). A thin layer of the dental cement was used to cover the entire exposed skull. For electrophysiological recordings, a ground screw was implanted in the skull above the cerebellum.

**Figure 1.**
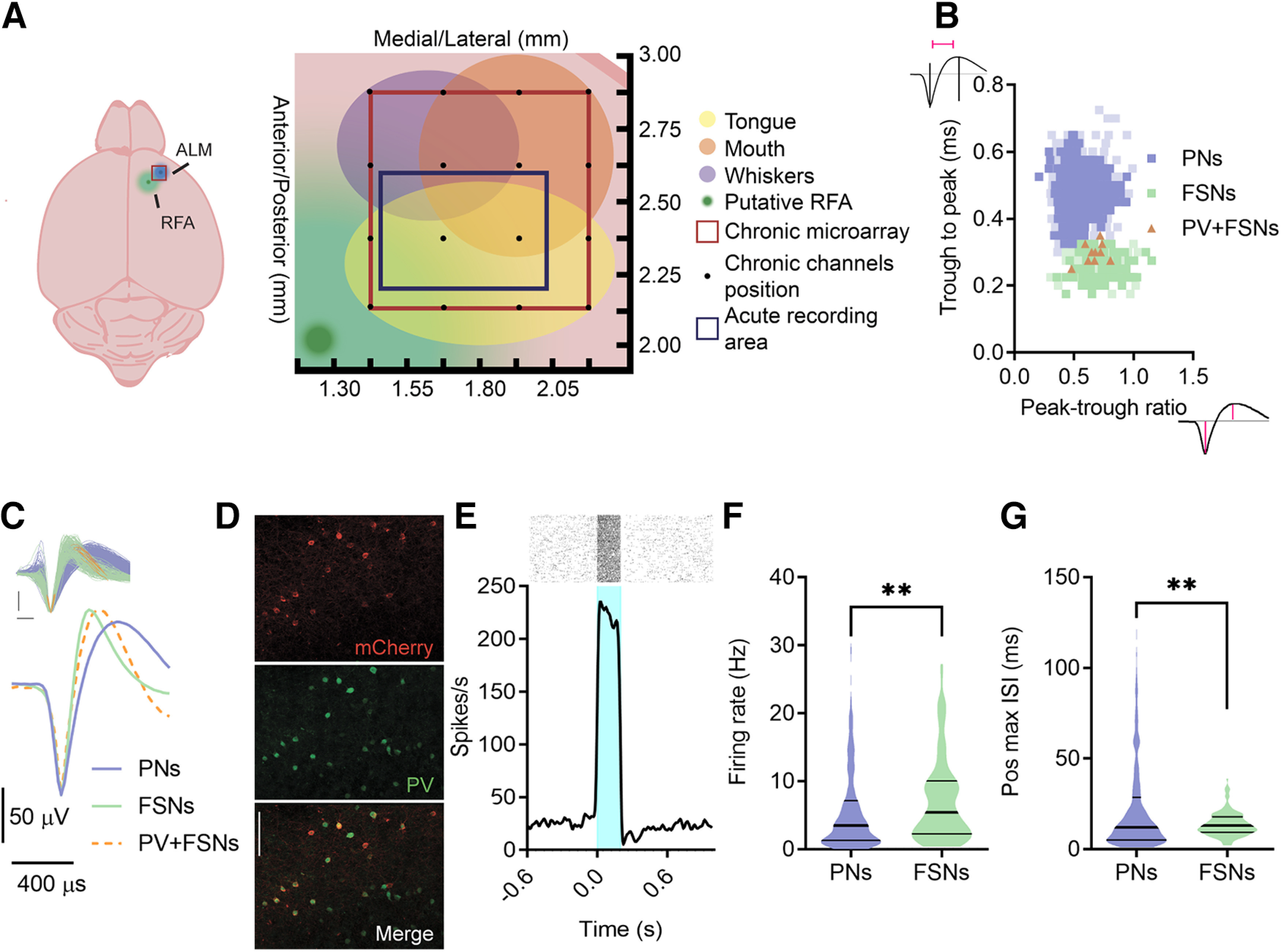
Electrophysiological recordings and PNs versus FSNs identification. ***A***, A schematic representation of the dorsal surface of the mouse brain showing the relative position of the putative ALM (blue) and the putative RFA (green). Magnification on the right shows the recording area (red square). The schematic diagram shows optogenetically identified movement representations in ALM, in particular, tongue-responsive area (yellow), whiskers-responsive area (violet), and mouth-responsive area (orange). The red square represents the chronic microarray position and the black dots indicate single microwires disposed in a 4 × 4 configuration. The blue rectangle shows the acute recording area. ***B***, Scatter plot of spike waveform parameters for all recorded units (*n* = 1452). The violet and green filled squares represent individual putative PNs (movement-related or not, violet and light violet, respectively) and FSNs (movement-related or not, green and light green, respectively), respectively. The orange filled triangles show spike shapes of individual PV+ FSNs (activated at short latency by light). ***C***, Average spike waveforms for all units, PNs, FSNs, and PV+ FSNs, aligned to minimum and normalized by trough depth. All waveforms are displayed in the inset (top). ***D***, Representative ALM portion of a PV-Cre mouse injected with the floxed ChR2-mCherry AAV (20×). MCherry reporter (red) indicates selective expression in parvalbumin-positive (PV) neurons stained by immunohistochemistry (green). Scale bar, 100 µm. ***E***, Representative raster plot and PSTH showing increased firing rate in response to 200-ms light pulses of an ALM ChR2+/PV+ FSNs recorded in a PV-Cre mouse injected with the floxed ChR2-mCherry AAV. ***F***, ***G***, Mean firing rate (***F***) and maximum position of interspike intervals (ISI; ***G***) of PNs and FSNs. K–S test, **p < 0.01.

For acute recordings, a recording chamber was built using dental cement (resin adhesive cement, Ivoclar Vivadent) and centered on the right ALM (1.8 mm lateral and 2.5 mm anterior to bregma; Fig. 1*A*). The skull over the recording area was covered by sterile low melting agarose type III (A6138, Sigma-Aldrich) and sealed with Kwik-Cast (WPI). On the day before the first acute recording session, six B6.Cg-Tg (Thy1-COP4/EYFP)18Gfng (ChR2) mice were anesthetized with ketamine (100 mg/kg) and xylazine (10 mg/kg) cocktail, the chamber cover removed and cortex was optogenetically stimulated following a grid with nodes spaced 500 μm. For each site, optogenetic stimulation (3 ms single pulses, 0.2 Hz) was delivered by means of PlexBright Optogenetic Stimulation System (PlexonInc) with a PlexBright LD-1 Single Channel LED Driver (PlexonInc) and a 473 nm Table-top LED Module connected to a 200 μm Core 0.39 NA optic fiber (ThorlabsInc). Movements of tongue/mouth were collected by a second experimenter, blinded to the stimulation coordinates. A small craniotomy (diameter, 0.5 mm) was then performed over sites where the larger tongue/mouth movements could be evoked. In wild-type mice, the craniotomy was performed in the same region of Thy1-ChR2 mice. Finally, the chamber was filled with agarose and sealed.

For chronic implants, a squared craniotomy (side: 0.8 mm) was made over the right ALM (1.8 mm lateral and 2.5 mm anterior to bregma), partially covering the rostral forelimb area (RFA; 1.2 mm lateral and 2 mm anterior to bregma; Fig. 1*A*). A planar multi shank 4 × 4 array (16 parallel microwires recording from their tips, Microprobes for Life Science) was positioned over the craniotomy and microwires were inserted into the cortex, up to ∼1000-µm depth to ensure better stability of the signal. Then, the craniotomy was covered with low melting agarose and the array fixed and embedded with dental cement (Super-Bond C&B and Paladur). Mice were allowed to awaken and then housed separately.

### Behavioral tasks and electrophysiological recordings

After recovering from surgery, mice were water restricted in their home cages, with food still available. Condensed milk was provided as a reward during the tasks and mice were also provided with water *ad libitum* for ∼1 h/d, following each recording session.

During the shaping phase, mice were placed in a U-shaped restrainer (3-cm inner diameter), head-fixed through the metal post cemented on their skull and habituated to lick reward drops, randomly provided by the experimenter through a feeding needle with no sensory cues enabling the animal to anticipate the delivery of the drop, which could be detected only by sniffing. Spontaneous licks were detected using a home-made piezoelectric licksensor implemented using Arduino. It has been set to precisely detect each time that the mouse tongue touches the spout, directly mounted on a piezoelectric sensor. We quantified the time between the first mouth opening and the first licksensor activation for each licking bout in three videos 120 fps) acquired during the recording sessions in three different animals. The licksensor did not alter neural trace with any artifact. Digital signals from the licksensor provided information about the licking movements directly to the recording apparatus.

Each shaping session lasted from 15 min up to 60 min for at least three consecutive days. Licking events were classified as either single or multiple licks. The start lick was defined as a movement that was not preceded (for at least 0.6 s) by any other licking event. A single lick was a start lick not followed by any other lick for at least 0.6 s; multiple licks were defined as start licks followed by at least two other consecutive licks (≤0.4 s among consecutive licks). Time intervals lasting for ≥1 s and distant at least 0.5 s from the end or the start of licking trials were considered as resting intervals and used as a baseline for the analysis of neural activity. To assess whether motivation of mice could influence the proportion of single and multiple licks, the frequency distributions of single and multiple licks were considered in our sessions. To normalize for different durations among sessions, each recording session was divided in 10 time windows and the number of single and multiple licks in each window has been counted; the relative frequency of single and multiple licks as a function of time along the session has been reported.

For identification of PV neurons in PV-cre mice, the site of AAV injection was illuminated with an optic fiber (200-µm Core 0.39 NA, Thorlabs). Optogenetic stimulation (50 0.2-s pulses, 0.2 Hz) was delivered by means of PlexBright System (Plexon) with a PlexBright LD-1 Single Channel LED Driver (Plexon) and a 473-nm Table-top LED Module. After spike sorting, PV-positive (i.e., FS) neurons were defined as neurons increasing their firing rate by 5 ms from the beginning of the blue light pulse (i.e., ChR2-positive neurons) and with a sustained activity for the entire stimulation length.

For the chronic recordings, in which forelimb-driven response was also assessed, head-fixed mice were shaped on a robotic platform, the M-Platform (Spalletti et al., 2014). Briefly, the M-Platform is composed of a linear actuator, a 6-axis load cell, a precision linear slide with an adjustable friction system and a custom-designed handle that is fastened to the left wrist of the mouse. The handle is screwed onto the load cell, which permits a complete transfer of the forces applied by the animal to the sensor during each session. The session started when the linear actuator moved the handle forward and extended the mouse left forelimb by 10 mm (full upper extremity extension). During recording sessions, the forepaw, contralateral to the implanted ALM/RFA, is maintained in a slightly isometric extended position; however, the animal voluntarily tries to pull the handle back to stay in a more comfortable posture, by retracting its forelimb (without any associated reward), and the force peaks exerted to attempt retractions are detected by the load-cell and offline aligned with neural signals.

In experiments with optogenetic inhibition of PV+ FSNs, 2 d after head-fixation implantation, mice were head-fixed and habituated to receive the liquid reward, delivered automatically, through an automatic peristaltic pump, 2 s after a 0.3-s acoustic-cue (4000 Hz). The pump was active for 0.3 s to deliver a drop of reward. After 2 d of habituation, a fiber optic was placed on their injected (right) ALM and the cue-signaled reward was randomly delivered in presence or absence of optogenetic stimulation. In a first set of mice (*n* = 2), the optogenetic stimulation consisted of a 1-s blue light train (3-ms pulses intermingled by 3-ms interpulse intervals, 473 nm), starting 1 s before the start of pump activation. In a second set of mice (*n* = 2), optogenetic stimulation was delivered 0.5 s before the start of pump activation. During the experiment, licking activity was detected through the licksensor and the frequency of licks was measured in a time window ranging from 0.5 s after start of reward delivery to 2.5 s later.

### Immunohistochemistry

Mice were perfused transcardially with PBS followed by 4% PFA. Brains were postfixed overnight and transferred to 30% sucrose PB solution before sectioning on a freezing microtome (Leica); 50-μm-thick coronal free-floating sections were processed using standard fluorescent immunohistochemical techniques: as primary antibodies we used: NeuN (1:1000, Millipore), GFAP (1:500, Dako), parvalbumin (1: 300, Synaptic System); as secondary antibodies, we used: anti-guinea pig AlexaFluor 488 (1:500, The Jackson Laboratory), anti-rabbit RRX (1:400, The Jackson Laboratory). MCherry and FusionRed signals were not amplified with immunostaining. Micrographs have been acquired using a fluorescence microscope (Zeiss).

### Single-unit recording and spike sorting

The electrophysiological data were continuously sampled at 40 kHz and bandpass filtered (300 Hz to 6 kHz), using a 16-channel Omniplex recording system (Plexon).

For acute recordings, a NeuroNexus Technologies 16-channel linear silicon probe with a single-shank (A1x16-3mm-50-177, 50-μm spacing among contacts) was slowly lowered into the ALM; the tip of the probe was placed at ∼1000-µm depth using a fine micromanipulator (IVM, Scientifica). The recording chamber was filled with sterile saline solution (NaCl 0.9%). Before the beginning of the recording, the electrode was allowed to settle for ∼10 min. For each animal, a number of one up to seven extracellular recording sessions were performed.

For chronic recordings, mice were recorded on up to 10–15 recording daily sessions per animal over a 15-d period.

The extracellular recording data were processed to isolate spike events by a spike sorting software (Offline Sorter v3.3.5, Plexon), using principal component analysis; events (spike-detection interval >1.0 ms) that exceeded a four SDs threshold above the background were sorted. The spike waveforms were aligned at global minimum and the artifact waveforms were removed. The single-unit clusters were manually defined.

### Data analysis

The recorded units were classified based on their average waveforms into putative pyramidal neurons (PNs) and putative fast-spiking neurons (FSNs). Two waveform parameters were used for the classification: the ratio between the height of the maximum peak and the initial negative trough, and the trough-peak time. A k-means clustering was applied. The clustering was verified by optogenetic tagging of PV-positive neurons.

The relation between single neuron activity and the events of the behavioral task was analyzed using MATLAB (MathWorks). Peristimulus time histograms (PSTHs) were built aligning spike events on the start lick, for both single and multiple licks, and on the onset of the force during forelimb pulling. Only intervals with stable unit activity were included and spikes were averaged over 0.05 s with 0.01-s steps. The PSTH covered a time window of 1 s, from 0.6 s before the starting event (lick or force onset) and 0.4 s after it. Responsive neurons were identified by comparing firing activity in the PSTHs with the mean firing rate (mfr) and an upper and lower threshold, calculated during resting periods (lasting ≥1 s, and distant from event trials ≥0.5 s). Bootstrapping was used to estimate the thresholds; lower and upper thresholds were, respectively, the 2.5th and 97.5th percentiles of the probability distribution function obtained during the resting intervals. A unit was considered responsive for the licking behavior or forelimb retraction when, for at least three consecutive bins (0.03 s), its firing rate went over (enhanced neurons) or under (suppressed neurons) the considered thresholds.

The onset of activity was defined as the first bin of the more than or equal to three consecutive bins above/below the upper/lower threshold; the time of the bin in which the firing rate (spikes per second) was maximum/minimum was considered as the peak time. To assess the influence of basal firing rate at rest (defined as above) on onset latency, a linear correlation was performed. To the same purpose, we pooled together FSNs and PNs with a licking-related activity and ordered them according to their resting firing rate (blinding the category they belong to); then, we compared the onset latency for each interquartile of FSNs and PNs.

The duration of the response was the number of bins above/below the upper/lower threshold. The intensity of activation was defined as:

$$\frac{areaabove/belowtheupper/lowerthreshold}{durationoftheresponse}.$$

Licking-related firing rate heat maps report normalized spiking activity of FSNs and PNs with enhanced licking-related activity. Firing rate has been normalized as follows: threshold firing rate (red) was set to zero, firing rate above threshold was normalized on the maximum and firing rate below the threshold was normalized on the minimum firing rate of each neuron, obtaining spiking activity ranging from −1 to 1.

Spatial selectivity for licking/forelimb activity was evaluated mapping the proportion of forelimb-, licking-related and licking/forelimb-related neurons among electrode positions of chronic arrays. Specifically, we considered total number of neurons modulated by licking L (i.e., “L+” + “L−”), forelimb F (i.e., “F+” + “F−”), and licking/forelimb LF (i.e., “L+/F+” + “L+/F−” + “L−/F+” + “L−/F−”).

### Information content

We measured the information content (Shannon, 1948) in all neurons with significant licking-related modulation (facilitated or suppressed). We considered the mean firing rate of each neuron about two different sets of conditions. Set 1: 0.8-s intervals centered at single licks (see above) versus rest, i.e., randomly selected 0.8-s intervals during which animals were at rest, distant at least 1.5 s from other licking or rest intervals. Set 2: 0.8-s intervals centered at the onset of multiple licks (see above) versus rest.

The mean firing rate (mfr) associated with each trial was measured over the whole window. The mutual information of summed firing rates (E, mfr) between mfr and each set of events E was computed as follows:

$$InformationofSummedFR(E,mfr)=\underset{e}{\sum }P(e)\underset{mfr}{\sum }P(mfr|e)*log2(\frac{P(mfr|e)}{P\left(mfr\right)}).$$

Where P (e) was the probability of the presentation of the specific event e, P (mfr) the probability over all trials and all conditions of the neuron to have the mean firing rate mfr in a given interval, P (mfr | e) the probability of the mean firing rate mfr to be associated to the event e. Mean firing rates were binned in N equipopulated bins, where N was the minimum value between the square root of the total number of trials and the number of unique values in the array of mean firing rates.

To reduce the bias in the estimation of the information because of the limited dataset, a quadratic extrapolation method was used (Panzeri et al., 2007). A statistically significant threshold was obtained bootstrapping 100 times (shuffling the conditions associated to each trial), and, for a major solidity, only neurons with an IC > 95th percentiles of the bootstrapped distribution, in at least one of the two combinations, were included, generating a subset of informative neurons.

We also calculated the information content over time: we considered 0.8 s before and 0.4 s after the first licking event, and we computed a local mean firing rate (Lmfr) over a moving average of 50 ms with 10-ms steps. Then, for each step we repeated the procedure described above. For this analysis we only used the subset of informative neurons described above.

For each recording session, we computed animal-wise the amount of information carried by summed firing rates of the recorded FSNs and PNs population. Each recording session has a different number of neurons and a different ratio between FSNs and PNs, for this reason, to be able to compare results from different recording sessions, the information of summed firing rates was computed considering N couples of neurons belonging to the same class for each recording. N was the minimum value between all the possible combinations of same-class-neurons and 40.

For each couple of neurons, information of summed firing rates was calculated with the following equation:

$$InformationofSummedFR\left(E,mfr1,2\right)=\underset{e}{\sum }P(e)\underset{mfr1,2}{\sum }P(mfr1,2|e)*log2(\frac{P(mfr1,2|e)}{P(mfr1,2)}).$$

Where *Information of Summed FR* (E, ISF 1,2) is the information given by the summed firing rates of neurons 1 and 2, *P* (e) was the probability of the presentation of the specific event e, *P* (*mfr* 1,2) the probability that the sum firing rate of the neurons to have the mean firing rate mfr 1,2 over all trials of all conditions, *P* (*mfr* 1,2 | e) is the probability of the mean firing rate (mfr 1,2) to occur during the event e.

We used the same bias correction method and the same statistically significant threshold of the previous analysis. Only couples with an information of summed FR > 95th percentiles of the bootstrapped distribution, in at least one of the two combinations, were considered.

We then normalized the *Information of Summed FR* (E, ISF 1,2) generating the information of summed FR index to the sum of the information contained in the mean firing rate of neurons 1 and 2 calculated separately with the following equation:

$$InformationofSummedFRIndex\left(1,2\right)=1-(\frac{ISFR(E,ISFR1,2)}{I\left(E,mfr1\right)+I(E,mfr2)}).$$

Where *Information of Summed FR Index* (1,2) is the normalized information carried by the sum of the firing rate of neurons 1 and 2, ISFR (E, ISFR 1,2) and I (E, mfr1) are defined above.

When *Information of Summed FR Index* (1,2) is equal to 0 it suggests that the information carried by the means of the two neurons are mostly independent, while higher values suggest that the information is more dependent.

### Statistical analysis

All data are expressed as mean ± SEM. Statistical tests were performed using GraphPad Prism 8.0 or SigmaPlot 12.0. Statistical significance was assessed using Wilcoxon test, Mann–Whitney test, one-way ANOVA, paired *t* test, and χ^2^ test, as appropriate. Cumulative distributions were tested using Kolmogorov–Smirnov (K–S) two-sample test. All statistical analyses were performed on raw data. The level of significance was set at *p < 0.05, **p < 0.01, ***p < 0.001.

## Results

### Electrophysiological identification of FSNs and PNs in head-fixed behaving mice

To clarify the causal role of FSNs in initiation and execution of spontaneous movements, we performed extracellular recordings within the premotor areas associated with licking and forelimb pulling movements, namely the ALM and RFA, respectively (Fig. 1*A*). We functionally identified the ALM by verifying, with optogenetic mapping in six Thy1-ChR2 mice, that its stimulation evoked mouth/tongue movements, whereas the identification of RFA was made based on previous literature (Alia et al., 2016; Spalletti et al., 2017; Svoboda and Li, 2018).

We extracellularly recorded neuronal activity from 1452 units with either an acutely inserted single shank, 16-channels silicon probe (*n* = 10 mice, *n* = 693 units) or a chronic 16-microelectrodes array (*n* = 3 mice; *n* = 759 units) from the ALM (and RFA, during chronic recordings). Spike detection and sorting were performed offline (Barthó et al., 2004; Mitchell et al., 2007; Niell and Stryker, 2010) to separate broad-spiking and narrow-spiking neurons, classified as PNs and FSNs, respectively (Fig. 1*B*,*C*).

To further validate the identification of FSNs, we performed extracellular recordings with optogenetic stimulation in mice expressing ChR2 selectively in parvalbumin-positive, fast-spiking cells (Tantillo et al., 2020; Fig. 1*D*,*E*). FSNs waveforms were included in the dataset before PNs/FSNs clustering: notably, all the optogenetically-tagged PV+ FSNs displayed a small trough to peak time and peak-trough ratio, coherently with their functional identification as putative interneurons, thereby confirming the reliability of our identification method. Moreover, narrow-spiking movement-related neurons displayed higher baseline activity (Fig. 1*F*) and shorter interspike interval (ISI; Fig. 1*G*) than broad-spiking neurons, consistent with the classification of the former as putative FSNs and of the latter as putative PNs.

### Activity of PNs and FSNs in the ALM during licking

**Figure 2.**
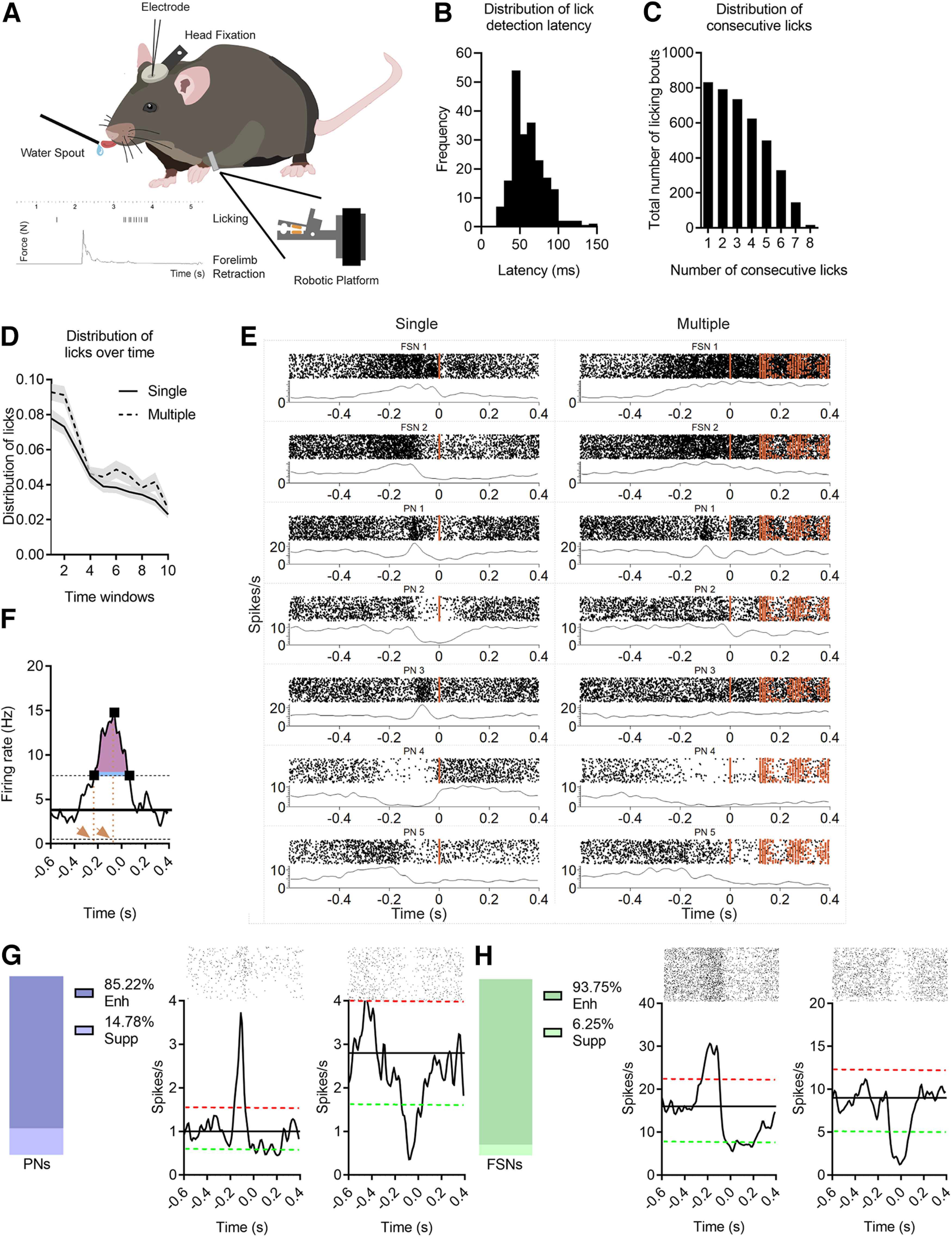
FSNs and PNs are modulated during spontaneous licking in mice. ***A***, Schematic representation of a head-fixed mouse in the behavioral setup. In the bottom left, a scale bar of the licking behavior and a forelimb force peak (N) are represented as a function of time (s). ***B***, Frequency distribution of latencies between licksensor activation (alignment event) and real onset of licking (first mouth movement detected in 120-fps videos). On average the movement onset started 61.9 ± 20.8 ms (mean ± SD) before licksensor detection. ***C***, Distribution of licking bouts based on the number of consecutive licks in a bout. Total number of licking events are reported as a function of the number of licks in the series (composed by 1–8 licks). ***D***, Average distribution of single and multiple licks among sessions. Each recording session is divided in 10 time windows. The number of single and multiple licks in each time window is reported as the relative frequency of single and multiple licks for each session. Repeated measures ANOVA, Group, *F* = 25.60, *p* < 0.001, Time, *F* = 44.58, *p* < 0.001, Group × Time, *F* = 0.77, *p* = 0.64. Data represented as mean ± shaded SEM. ***E***, Seven examples of ALM neurons during licking task, in single (left column) and multiple (right column) licks. Spike rasters and PSTHs are reported for two FSNs and five PNs in both single and multiple licks. Averaging window, 100 ms. Orange squares represent licks (i.e., tongue touches) for each trial. ***F***, Representative peristimulus time histogram. The black line represents the average firing rate calculated during resting periods, black dotted lines the upper and lower threshold. The three black squares indicate the first, the maximum and the last point over the threshold. The orange dotted lines and the orange arrows indicate the onset of the activity and the peak time, respectively. The blue line shows the duration of the activity, representing the time over the threshold. The pink area is the area above the threshold. The intensity of activation is defined as the pink area divided by duration of the activity. ***G***, ***H***, Proportion of all responsive putative PNs: enhanced, violet, or suppressed, light violet (***G***) and putative FSNs: enhanced, green, or suppressed, light green (***H***) during the licking activity. On the right, representative examples of raster plots and corresponding PSTHs showing enhanced (left) and suppressed (right) neurons. The red dotted lines represent the upper thresholds, the green dotted lines the lower ones, the black line is the mean baseline firing rate. Time = 0 corresponds to the first lick, not preceded by other licks for at least 0.6 s. PNs suppressed versus FSNs suppressed, *z*-test, *z* = 1.65, *p* = 0.09.

Water-restricted, head-fixed mice were allowed to lick drops of liquid reward spontaneously (not signaled by any cue), available through a drinking spout, centered in front of the animal and detecting licking events through a piezo-based licksensor (Fig. 2*A*). To quantify the latency between the onset of the licking movement and the licksensor activation, we analyzed 120-fps videos of a subset (*n* = 3) of the recorded sessions (in different animals) and measured the number of frames interposed between mouth opening onset and licksensor switching. We found a latency of 61.9 ± 20.8 ms (mean ± SD; Fig. 2*B*). Offline, we categorized licking bouts based on their lick numerosity. We found bouts composed of up to eight consecutive licking events (categories including from six to eight events were less represented; Fig. 2*C*). To assess whether neural activity reflects the sequential encoding of each motor chunk in a licking bout or whether it is associated with the whole sequence of movements, we analyzed isolated “single” (one lick) and “multiple” (more than or equal to three consecutive licks) bouts (see Materials and Methods). Because of the spontaneous nature of our task, we checked the time distribution of single and multiple licks over the recording sessions to control motivation effects on licking behavior. We found a simultaneous gradual dispersion over time, consistent with the increasing satiation of animals, but importantly, we did not find differences in the distribution of single and multiple licks along the session (not significant Group × Time interaction; Fig. 2*D*)

Peristimulus time histograms (PSTHs) were created by aligning the spiking activity of each neuron to the first tongue touch of each licking bout (see example neurons in Fig. 2*E*). For each neuron, the mean firing rate was compared with a threshold (Fig. 2*F*; Materials and Methods) to identify significantly responsive neurons. Overall, in both acute and chronic recordings, we found 624 out of 1452 units (36%) significantly modulated during movement, whereas the remaining were not significantly modulated during motor activity. Out of 624 movement-related neurons, 251 (203 putative PNs, 48 putative FSNs; Table 1) were recorded in the first set of experiments with acutely inserted silicon probes in the ALM. The majority of putative PNs showed enhanced firing rate during licking, and only 15% of them exhibited a suppressed discharge during licking epochs (Fig. 2*G*). Among FSNs, the proportion of licking-suppressed neurons was lower (∼6%; Fig. 2*H*).

**Table 1.** Total number of recorded units during acute and chronic experiments

	Total recorded units	Modulated units	PNs	FSNs
Acute Exp	693	251	203	48
Chronic Exp	759	373	313	60

The modulated PNs and FSNs are also reported.

**Figure 3.**
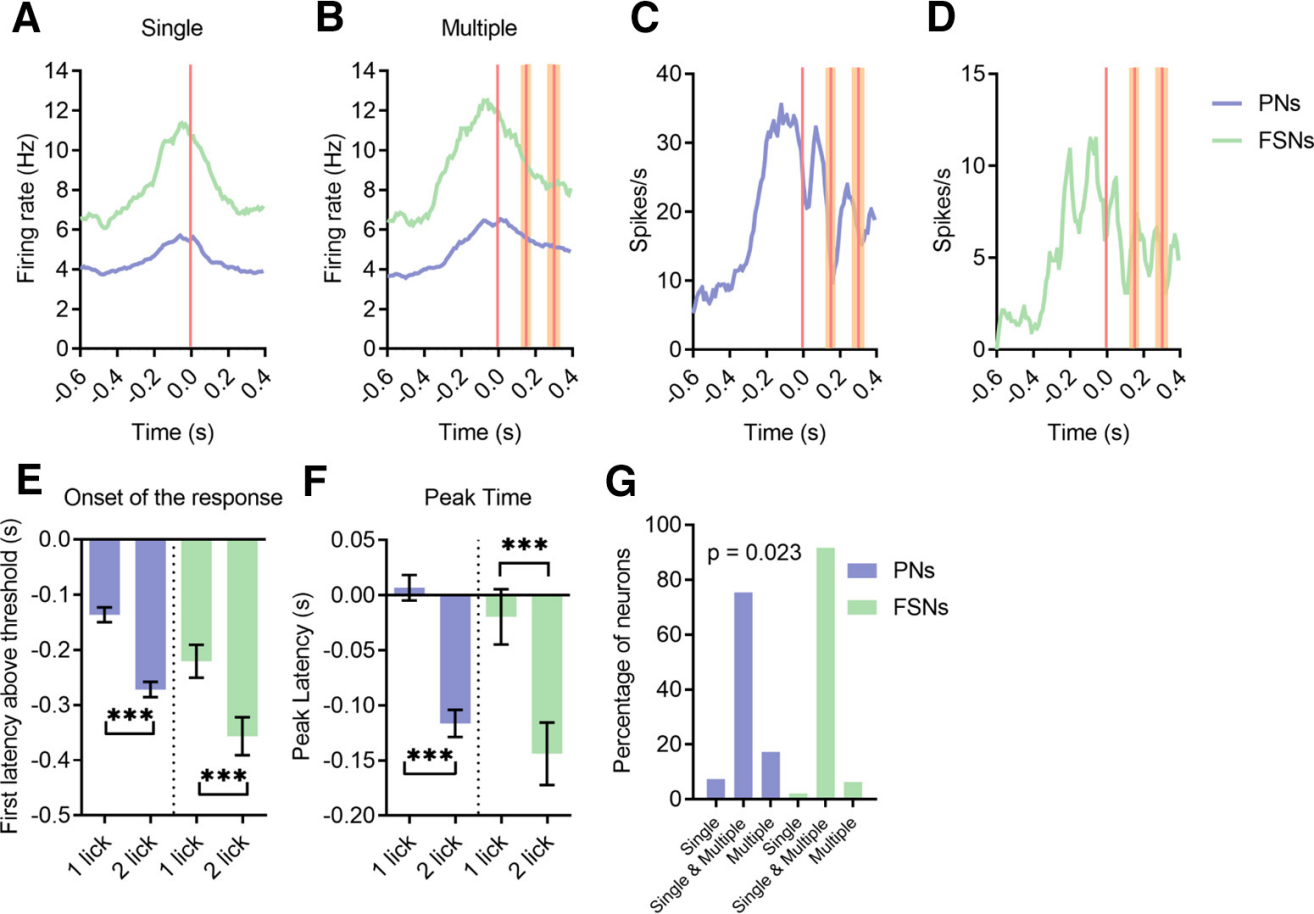
FSNs and PNs in ALM encode entire licking bout during single and multiple licks. ***A***, ***B***, Average PSTHs for all PNs (violet) and FSNs (green) in a 1-s window (0.6 s before and 0.4 s after the licking event) during single (***A***) and multiple (***B***) licks. Time 0 > (vertical red bars) corresponds to the first lick. The second and the third bars in multiple licks indicate the mean position (±SEM, orange shades) of the second and the third licks, respectively. ***C***, ***D***, Representative PSTHs of a PN (***C***) and a FSN (***D***) showing lick-by-lick modulation in a 1-s window (0.6 s before and 0.4 s after the licking event) during multiple licks. Time 0 (vertical red bars) corresponds to the first lick. The second and the third bars in multiple licks indicate the mean position (±SEM, orange shades) of the second and the third lick, respectively. ***E***, ***F***, Histograms of the onset of the response (***E***) and the peak time (***F***) of PNs and FSNs obtained aligning PSTHs to the first or the second lick of a licking bout. Wilcoxon test, ***p < 0.001. ***G***, Percentage of PNs and FSNs responsive to both single and multiple licks, or selective for single or multiple licks. χ^2^ test, χ^2^_(1)_ = 5.18, *p* = 0.023.

Both PNs and FSNs showed the maximum response modulation at the licking bout initiation, even in the case of multiple licks, suggesting that their activity could contribute to the entire sequence rather than the generation of each individual lick. This can be clearly appreciated by building mean PSTHs for the two classes of neurons (Fig. 3*A*,*B*). We found that in multiple licks, by aligning neuronal spiking on the first lick of a bout, the PSTH displayed a unique peak before the beginning of the series (Fig. 3*B*, average of all PNs and FSNs) while only a small fraction of the recorded units (2% of FSNs and 5% of PNs; Fig. 3*C*,*D*) showed a series of recurrent peaks time locked with each licking event. Consistently, comparing mean PSTHs aligned on the first or the second lick in the series, both onset of the response (Fig. 3*E*) and peak of activity timing (Fig. 3*F*) were shifted backward of ∼0.150 s relative to the alignment on the first lick, which corresponds to the typical time lag between subsequent licking events in a series. These data support the hypothesis that neuronal discharge of both PNs and FSNs in the ALM is mainly related to start the execution of the entire licking bout rather than the execution of individual licks in a series.

Next, we assessed the possible difference between PNs and FSNs in the encoding of licking bouts made of single or multiple licks, starting from the evidence that individual neurons can discharge differently prior and during these types of behavior (see neuron examples in Fig. 2*E*). We plotted the percentages of PNs and FSNs modulated exclusively during single licks, multiple licks, or both (Fig. 3*G*). The comparison reveals that the majority of both FSNs and PNs discharge for licking bouts regardless of the number of lick events constituting the bout (either one or more than two licks), and that this behavior is prevalent among FSNs relative to PNs. These data indicate that although single and multiple licks can be encoded differently, FSNs have a broader tuning than PNs.

### FSNs show earlier and more sustained activation than PNs during licking

**Figure 4.**
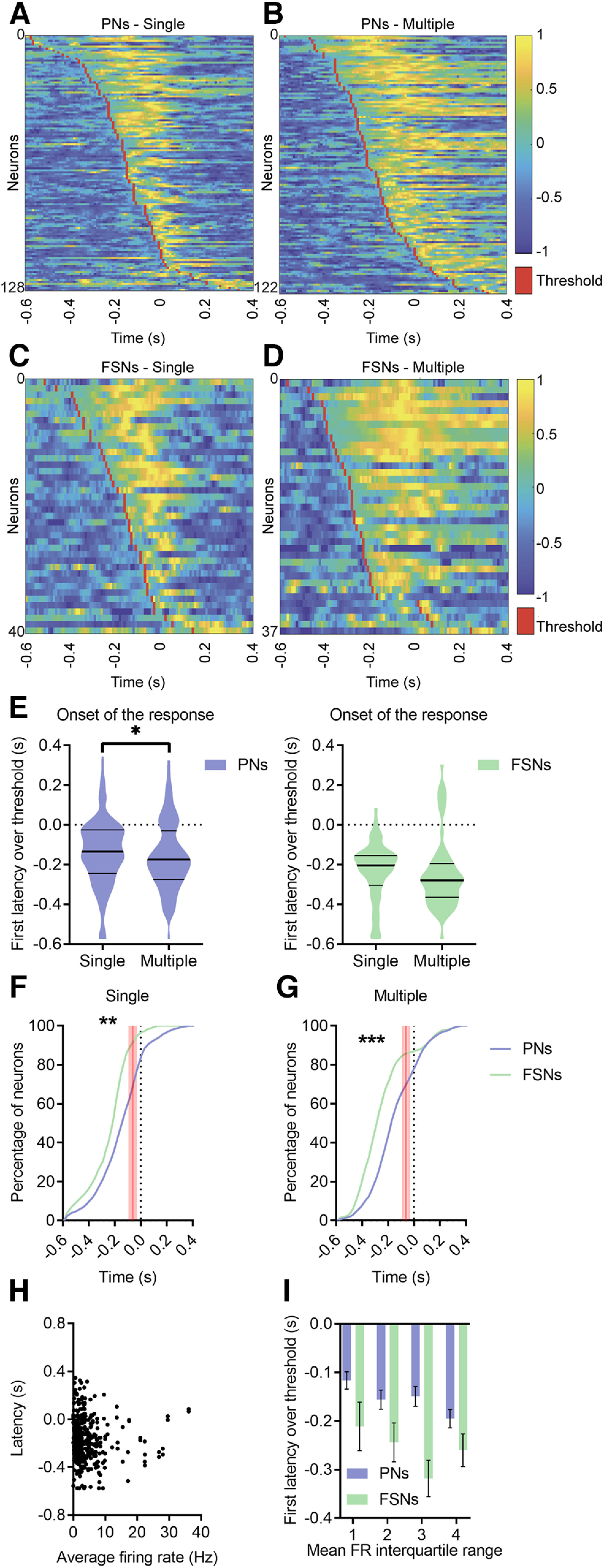
FSNs show earlier activation than PNs during licking in single and multiple licks ***A–D***, Heat maps for all positively modulated PNs (***A***, ***B***) and FSNs (***C***, ***D***) ordered by the onset of the response, during both single and multiple licks. Normalized spiking activity is reported, ranging from −1 to 1. Threshold firing rate (red) was set to zero, firing rate above threshold is normalized on the maximum and the one below threshold on the minimum for each neuron. ***E***, Violin plots of onset of the response, defined as the first latency above or below the thresholds on PSTHs, for PNs (left) and FSNs (right), during single and multiple licks (always aligned to the first lick). Wilcoxon test, *p < 0.05. ***F***, ***G***, Cumulative distribution of the onset of the response for all PNs and FSNs during a single isolated lick (***F***) or multiple licks (***G***). The red shaded lines indicate the confidence interval (61.9 ± 20.8 ms) of movement initiation, the first mouth movement before licksensor activation. K–S test, single, **p = 0.001, multiple, **p < 0.001. ***H***, Correlation between mean firing rate and onset latency in all recorded neurons. Rho = −0.0650, *p* = 0.197. ***I***, All neurons were pooled and then grouped in interquartile ranges, according to their resting firing rate. Latency of activation of FSNs and PNs was then compared for each interquartile. A two-way ANOVA showed that factor neuronal type explained a large fraction of variance (*F* = 19.9, *p* < 0.0001) while the factor interquartile did not (*F* = 2.18, *p* = 0.08). There was no interaction between the factor interquartile and the factor neuronal type (*F* = 0.33, *p* = 0.8). Data are represented as mean ± SEM.

We next investigated PNs and FSNs firing activity during single and multiple licks (Fig. 4*A–D*). First, we analyzed the onset of the (enhanced or suppressed) response, revealing that most of the recorded neurons exhibit a significant modulation before movement onset, independently from the forthcoming licking strategy (Fig. 4*E*), but onset of PNs discharge occurred earlier in relation to multiple than single licks, whereas FSNs fired ∼0.1 s earlier than PNs but with no significant difference between multiple and single licks. A cumulative distribution curve of the onset for individual neurons (Fig. 4*F*,*G*) clearly indicate an earlier recruitment of FSNs. This early activation of FSNs was not a by-product of their overall higher firing rate with respect to PNs, since there was no correlation between resting firing rate and onset latency in licking-responsive neurons (ρ = −0.065, *p* = 0.197; Fig. 4*H*). Moreover, we did not find any effect of baseline firing rate in explaining differences between PNs and FSNs onset, since comparison of onset latency between PNs and FSNs with similar firing rate (same interquartile), showed an effect for the neuronal type but neither for the firing rate class, nor for the interaction (Fig. 4*I*).

**Figure 5.**
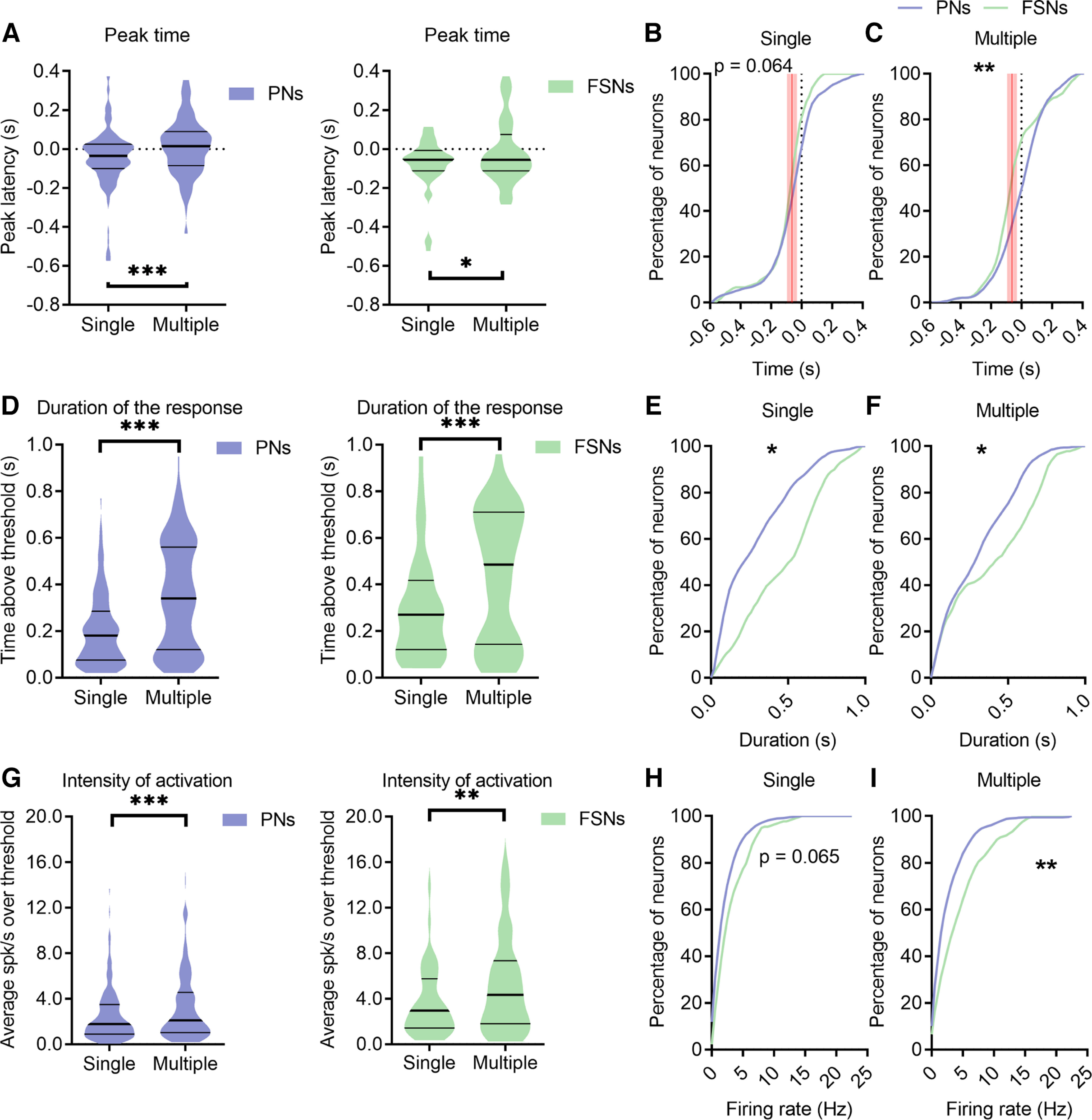
FSNs show more sustained activation than PNs during licking. ***A***, Violin plots of the peak time of PNs (left) and FSNs (right) during single and multiple licks (always aligned to the first lick). The peak discharge is significantly delayed for both PNs and FSNs during multiple licks. Wilcoxon test, *p < 0.05, ***p < 0.001. ***B***, ***C***, Cumulative distribution of the peak time for all PNs and FSNs during a single isolated lick (***B***) or consecutive multiple licks (***C***). The red shaded lines indicate the confidence interval (61.9 ± 20.8 ms) of movement initiation, the first mouth movement before licksensor activation. K–S test, single, *p* = 0.064, multiple, **p = 0.0063. ***D***, Violin plots of the duration of the response of PNs (left) and FSNs (right), during single and multiple licks. Wilcoxon test, ***p < 0.001. ***E***, ***F***, Cumulative distribution of the duration of the response for all PNs and FSNs during a single isolated lick (***E***) and consecutive multiple licks (***F***). K–S test, single, *p = 0.0158, multiple, *p = 0. 0269. ***G***, Violin plots of the intensity of activity of PNs (left) and FSNs (right), during single and multiple licks. Wilcoxon test, **p < 0.01, ***p < 0.001. ***H***, ***I***, Cumulative distribution of the intensity of activation for all PNs and FSNs during a single isolated lick (***H***) and multiple licks (***I***). K–S test, single, *p* = 0.065, multiple, **p = 0.0051.

Then, we examined the timing of the peak of activity (or suppression) for each neuron. In multiple licks, the average peak time was delayed for both PNs and FSNs (Fig. 5*A*). Cumulative distributions of the peak latency are reported in Figure 5*B*,*C*. A robust statistical difference between PNs and FSNs was present for multiple licks: specifically, one third of PNs reached their maximum firing rate before the onset of the licking bout, whereas about half of FSNs had their peak of activity before licking onset (Fig. 5*C*). Next, we explored the duration of neuronal response, which was greater for both PNs and FSNs when mice performed multiple versus single licks (Fig. 5*D*). Interestingly, the response duration was overall longer in FSNs during both single and multiple licks as compared with PNs (Fig. 5*E*,*F*).

Similar results were obtained by analyzing the magnitude of the activation of the two neuronal classes. During multiple licks, both PNs and FSNs showed greater discharge than during a single lick (Fig. 5*G*). It is worth noting that, as reported above (Fig. 2*D*), although we confirmed that motivation has an effect on the total number of lick events over time, no difference between the rate of single versus multiple licks was observed, allowing us to safely exclude a role of satiation state of the animals in causing the electrophysiological differences between single and multiple licks. Furthermore, the FSNs displayed a higher activity relative to PNs, which was more evident in multiple than in single licks (Fig. 5*H*,*I*).

Altogether, these findings show that FSNs have an earlier and sustained firing activity with respect to PNs during the movement, independently of the licking strategy, i.e., single or multiple licks, which nevertheless are coded by differential response patterns of both PNs and FSNs in terms of onset, peak discharge, duration and magnitude of their firing activity.

### Information content of firing rate

We next computed, for all the previously identified responsive neurons, the mutual information between the firing rate and the behavioral states (i.e., rest, single lick and multiple licks; see Materials and Methods). The fraction of informative neurons was 0.74 for FSNs and 0.63 for PNs. Within the subset of informative neurons, FSNs carried vastly more information than PNs about the onset of both single (0.130 bits, FSNs; 0.074 bits, PNs) and multiple licks (0.221 bits, FSNs; 0.140 bits, PNs).

**Figure 6.**
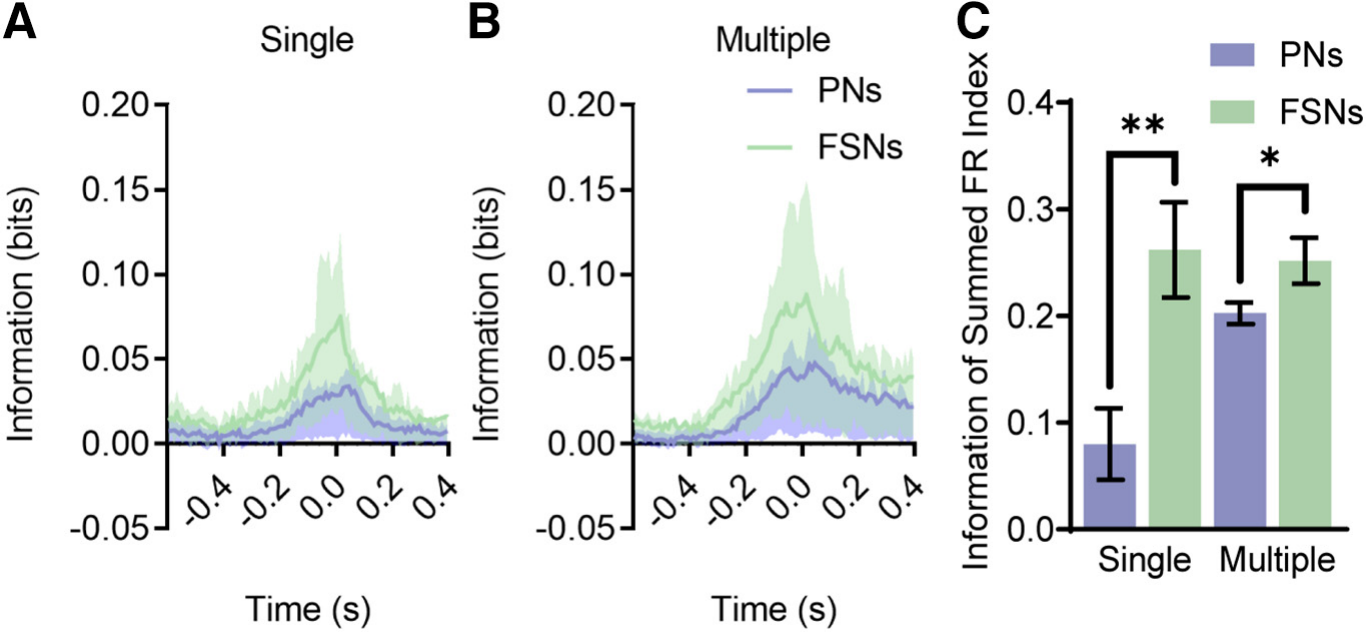
FSNs convey a considerable amount of information and before PNs activation. ***A***, ***B***, Information carried by firing rate of PNs (violet) and FSNs (green) about the presence of single (***A***) and multiple (***B***) licks. Information is computed over 0.05 s bins (with a sliding time window of 0.01-s width) in a 1-s window (0.6 s before and 0.4 s after the licking event). Lower and higher shades represent, respectively, the 25th and 75th percentiles. Wilcoxon test, *p* < 0.001. ***C***. Information of summed firing rate index for couple of PNs and FSNs of the same recording session for both single and multiple licks. Mann–Whitney test, *p < 0.05, **p < 0.01. Data are represented as mean ± SEM.

Coherently with an earlier onset of the response, FSNs information content ramped up earlier than that of PNs (Fig. 6*A*,*B*). Information carried by FSNs became three SD larger than baseline for at least two consecutive bins ∼0.05 s earlier than PNs. Comparing single licks and rest, the information exceeded the threshold 0.25 s before lick detection in FSNs and 0.2 s in PNs. The peak of information was reached at the tongue touch in FSNs and 0.03 s later in PNs. Multiple licks versus rest yielded similar results: the information exceeded the threshold 0.33 s before the first licking event in FSNs and 0.27 s in PNs; the peak was reached 0.02 s after the event in FSNs and 0.05 s in PNs. Temporal dynamics of the information content was similar to the FSNs and PNs features shown by the results in previous section (Fig. 5) and global PSTHs (compare Figs. 6*A*,*B* and 3*A*,*B*).

We then computed the animal-wise amount of information carried by the summed firing rate of the recorded FSNs and PNs population and found that FSNs carried more redundant information. The Information of summed firing rate index (see Materials and Methods) is significantly higher for FSNs than for PNs (mean 0.26 for FSNs; 0.08 for PNs, single licks vs rest; mean 0.25 for FSNs; 0.20 for PNs, multiple licks vs rest; Fig. 6*C*).

Overall, these results suggest that the local firing rate of FSNs conveys a considerable amount of information before PNs activation, further supporting the idea that a robust and coherent inhibitory activity might be important before and during the movement.

### Layer-specific responses of PNs and FSNs

**Figure 7.**
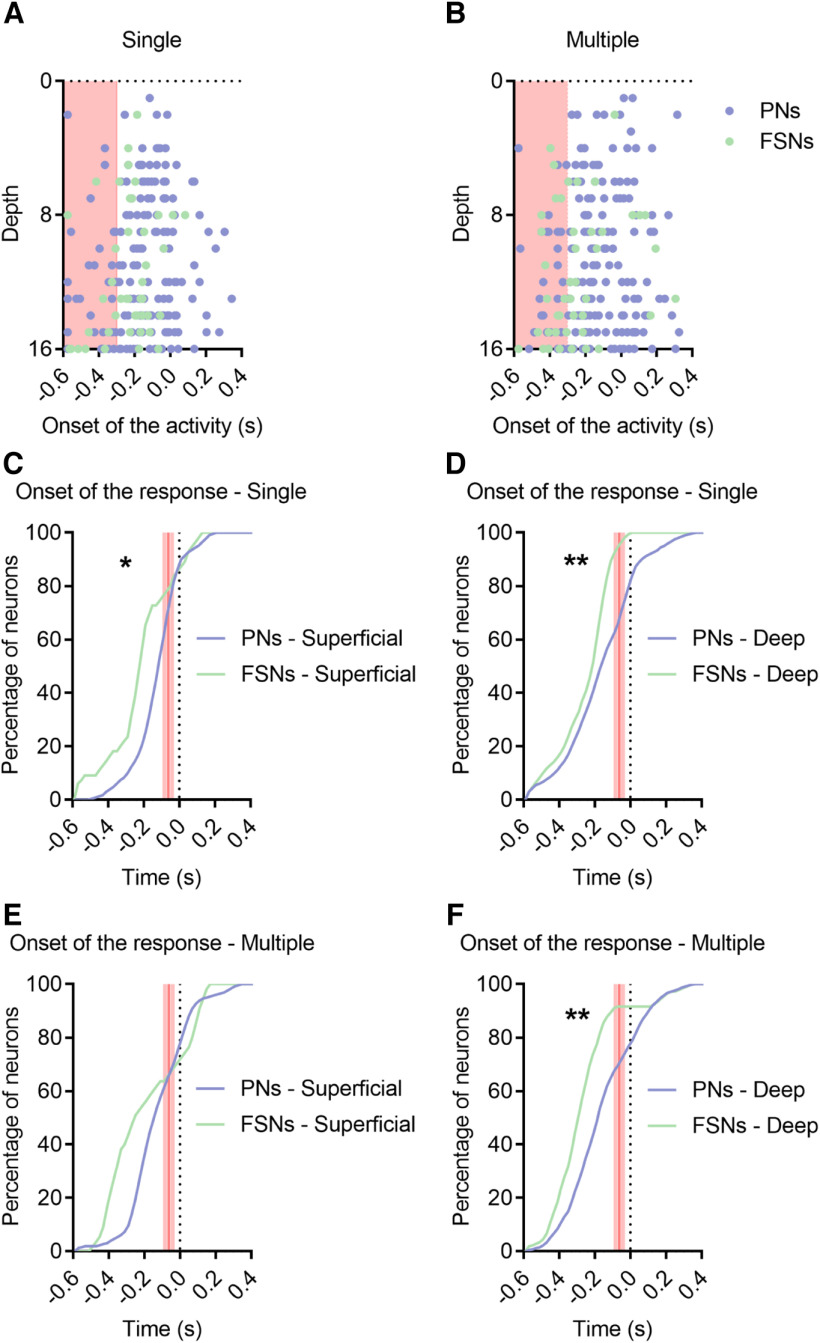
Relative temporal firing dynamics between FSNs and PNs is conserved over layers ***A***, ***B***, PNs (violet) and FSNs (green) depth distribution (across sixteen channels probe) of the onset of the activity in a 1-s window (0.6 s before and 0.4 s after the licking event) during single (***A***) and multiple (***B***) licks. Red-shaded part indicates –0.6 / –0.3 s time window. ***C***, ***D***, Cumulative distribution of the onset of the response for superficial (***C***) and deep (***D***) PNs and FSNs during a single isolated lick. The red shaded lines indicate the confidence interval (61.9 ± 20.8 ms) of movement initiation, the first mouth movement before licksensor activation. K–S test, single, superficial, *p = 0.0198. K–S test, single, deep, **p = 0.0062. ***E***, ***F***, Cumulative distribution of the onset of the response for superficial (***E***) and deep (***F***) PNs and FSNs during multiple licks. Red shaded lines as in C, D. K–S test, multiple, superficial, *p* = 0.150. K–S test, multiple, deep, **p = 0.0019.

Linear probes allowed us to investigate the laminar distribution of recorded neurons. Specifically, units were classified as superficial (channels 1–8, ∼<600 µm in depth) or deep (channels 9–16, ∼>600 µm in depth). In our sample, ∼25% of PNs and FSNs were recorded from superficial layers. Figure 7*A*,*B* reports the onset of activity for each recorded unit as a function of depth (i.e., channel number). While the average response onset of FSNs precedes the one of PNs (consistently with Fig. 4*E–G*), a small proportion of PNs (especially in deep layers) appear to increase their firing rate earlier, simultaneously with FSNs. Furthermore, firing activity appears to start earlier in deep relative to superficial layers (Fig. 7*A*,*B*, red-shaded part of panels).

Considering onset latency separately in deep or superficial PNs and FSNs, we substantially confirmed findings obtained over all cortical layers. In fact, FSNs activity starts significantly earlier than PNs activity in both superficial (Fig. 7*C*) and deep (Fig. 7*D*) layers during single licks. During multiple licks, the activity of FSNs starts significantly earlier than that of PNs in deep (Fig. 7*F*), but not in superficial (Fig. 7*E*) layers.

These results suggest that initial activity mostly begins in deep layers of ALM (Chen et al., 2017), and involves both FSNs and PNs.

### Direct comparison of the neuronal responses of PNs and FSNs during two motor acts

**Figure 8.**
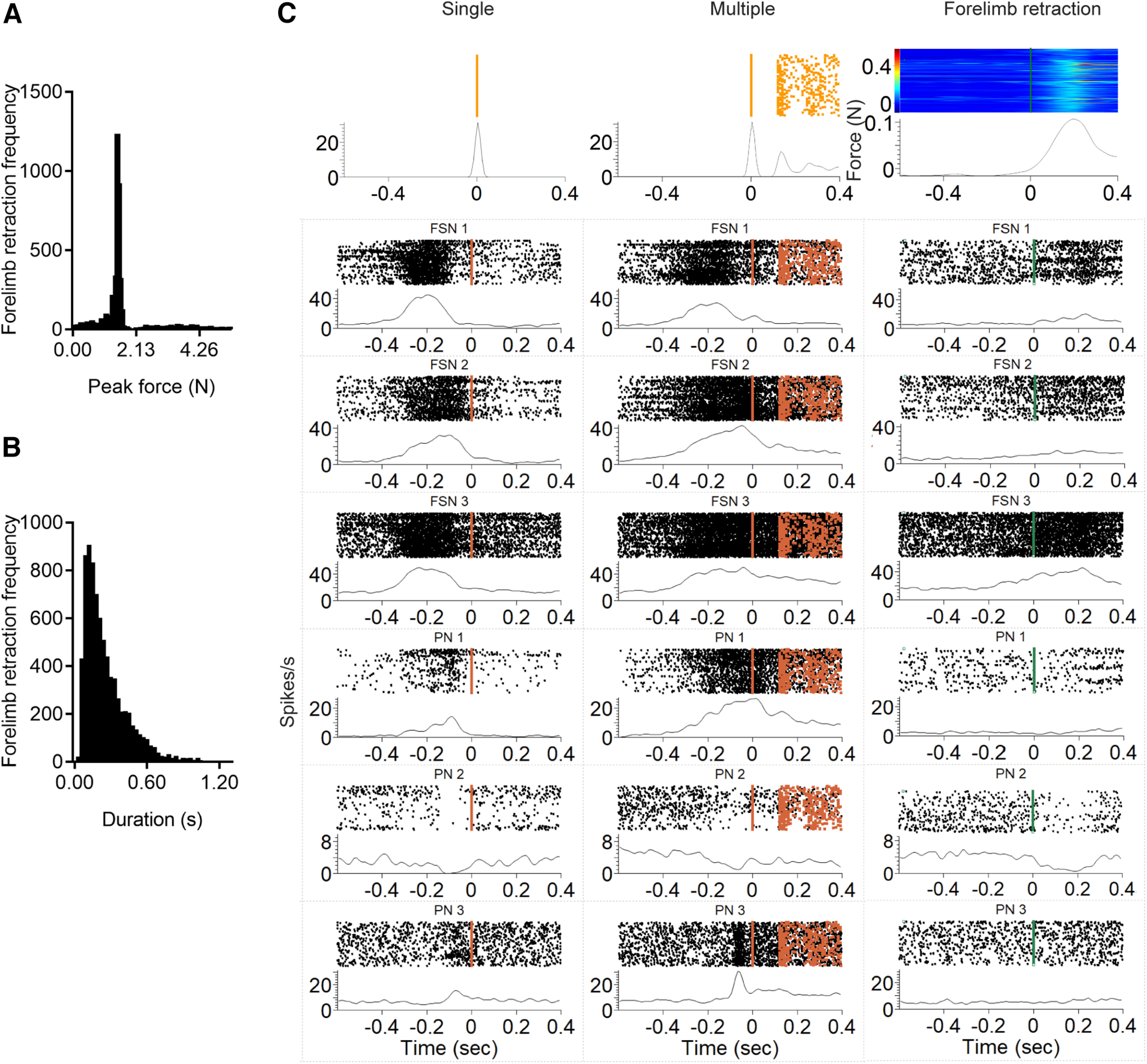
ALM FSNs and PNs are modulated during spontaneous forelimb pulling ***A***, ***B***, Frequency distribution of force peaks (***A***) and duration (***B***) of forelimb retraction. Averaging windows, 0.03 N (force peaks, ***A***) and 500 ms (duration, ***B***). ***C***, Six examples of ALM and RFA neurons, three FSNs and three PNs, are reported during licking task, in single (left column) and multiple licks (central column), and during forelimb retraction (right column). For each panel, in the top, spike rasters and PSTHs are reported for each neuron in all the three conditions; in the top right of the figure, the force during forelimb retraction is reported. Averaging window, 100 ms. Orange squares represent licks for each trial, green triangles the forelimb retraction for each trial.

Early and sustained inhibition by FSNs during licking may be a general mechanism that contributes to action selection before movement onset, regardless of the effector to be used for acting. To test this hypothesis, we compared the activity of a set of FSNs and PNs, recorded in head-fixed mice during two types of motor tasks, i.e., a forelimb retraction task in addition to the licking task. We took advantage of a robotic platform (M-Platform; Spalletti et al., 2017; Allegra Mascaro et al., 2019), which allows mice to perform several trials of spontaneous forelimb pulling (without associated rewards), resulting in force peaks, recorded by a load cell embedded in the M-Platform (Fig. 2*A*). Distribution of maximum force and duration of force peaks in our dataset were reported in Figure 8*A*,*B*. Neurons' discharge was aligned to the onset of force peaks (Spalletti et al., 2014; Pasquini et al., 2018; Fig. 8*C*). Animals were also allowed to perform spontaneous licking within the same experimental session, albeit in different epochs. In the following sections, we describe the neuronal discharges during pulling and multiple licking events (i.e., spaced by >0.6 s from any type of movement).

**Figure 9.**
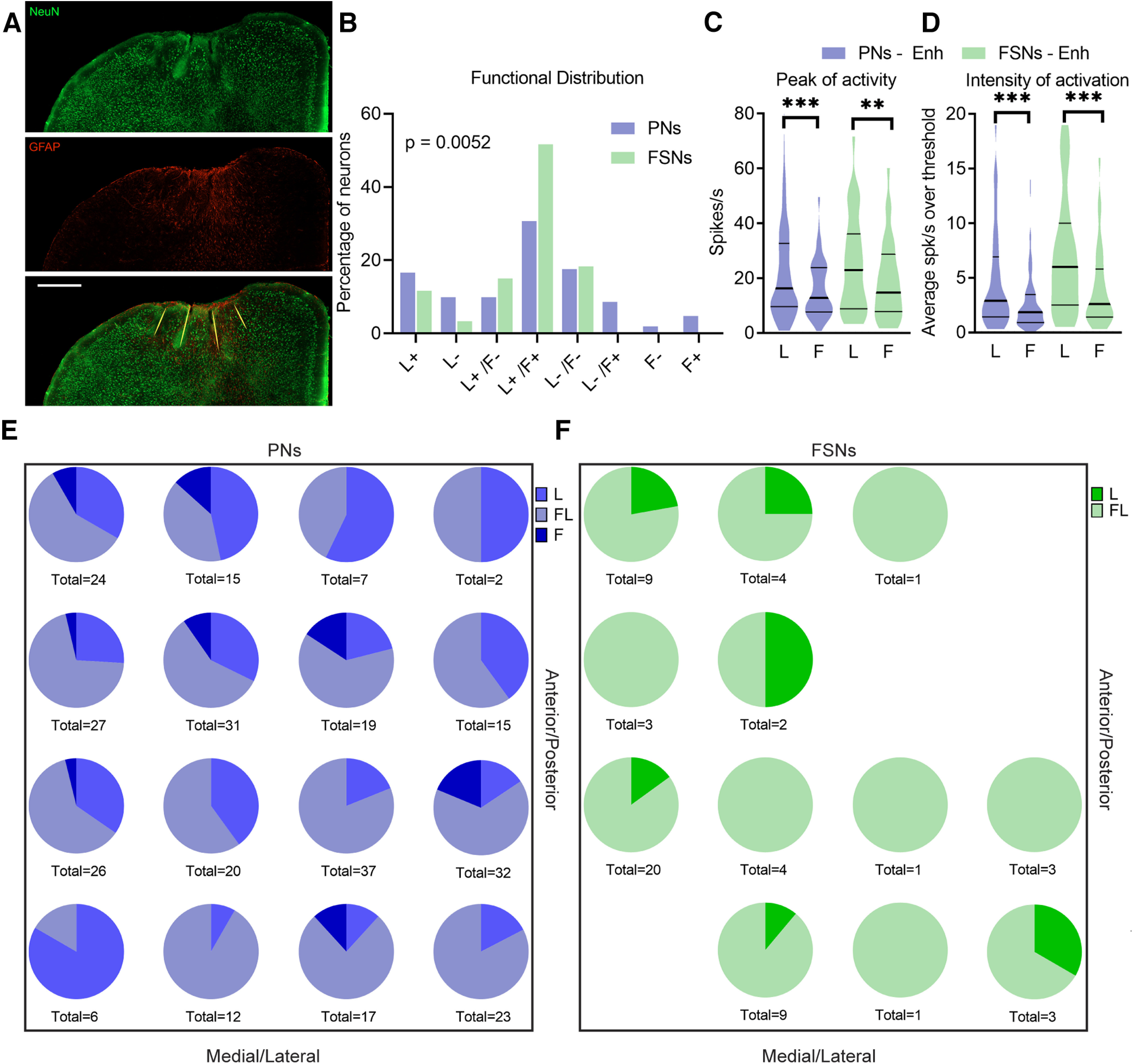
FSNs exhibit lower selectivity than PNs for licking behavior and forelimb retraction. ***A***, Representative image of four microwires traces after removal of implanted chronic electrodes (20× tile; scale bar, 500 µm). The immunostaining against the neuronal marker (NeuN, green) and reactive astrocytes (GFAP, red) show the site of microwires insertion (yellow lines) in a coronal section of the ALM. ***B***, Functional distribution of neurons responsive for licking (L), forelimb pulling (F) or both of them (LF), classified as enhanced (+) or suppressed (−) by the movement. χ^2^ test, χ^2^_(7)_ = 20.19, *p* = 0.0052. ***C***, ***D***, Peak of activity and intensity of activation for all PNs (violet) and FSNs (green) increasing their discharge during both forelimb retraction (F) and multiple licks (L) tasks. Peak of activity, paired *t* test, PNs, Enh, *t*_(1,91)_ = 3.97, ***p = 0.0001, FSNs, Enh, *t*_(1,30)_ = 3.17, **p = 0.0035. Intensity of activation, paired *t* test, PNs, Enh, *t*_(1,91)_ = 4.47, ***p < 0.0001, FSNs, Enh, *t*_(1,30)_ = 4.07, ***p = 0.0003. ***E***, ***F***, Proportion of PNs (***E***) and FSNs (***F***) selective for forelimb pulling, multiple licking or both, among electrode positions over the region covered by the 4 × 4 chronic microelectrode array in the ALM and the portion of sampled RFA.

For these experiments we employed a planar 4 × 4 chronic array, centered on the ALM but exceeding the boundary with the adjacent RFA (Tennant et al., 2011; Alia et al., 2016; Figs. 1*A*, 9*A*). To allow greater stability during the recordings, electrode contacts were positioned in deep layers. We isolated *n* = 373 units (PNs, *n* = 313; FSNs, *n* = 60; mice, *n* = 3; Table 1), which were responsive to licking, pulling, or both.

We found a great proportion of neurons whose discharge was suppressed during licking, higher with respect to previous data collected in acute recordings. Specifically, 37% of PNs, whose discharge was modulated during licking behavior showed movement-related suppression of their discharge; a similar proportion (40%) of PNs responsive for forelimb retraction were also suppressed. For FSNs, the percentages of suppressed neurons were similar (39.1%) for forelimb retraction, and lower (20.3%) for licking. These data suggest that pyramidal neurons as well as FSNs located in deep layers are particularly susceptible to movement-related suppression. Therefore, we analyzed enhanced and suppressed neurons separately (Table 2).

**Table 2.** Number of neurons in different functional classes

	Lick Enh	Lick Supp	Lick Enh/FP Supp	Lick Enh/FP Enh	Lick Supp/FP Supp	Lick Supp/FP Enh	FP Supp	FP Enh
PNs	52	31	31	96	55	27	6	15
FSNs	7	2	9	31	11	–	–	–

Lick, licking; FP, forelimb pulling; Enh, enhanced; Supp, suppressed.

### Lower motor selectivity for licking and forelimb movement in FSNs than PNs

Neuronal selectivity for each type of movement (i.e., multiple licks vs pulling) was assessed comparing distribution of FSNs and PNs whose activity was modulated selectively during multiple licks (L), forelimb pulling (F), or both (LF). In particular, we subdivided the recorded units into different functional classes, according to the movement-induced modulation of their discharge. Specifically, neurons responsive to only one type of movement were classified as enhanced/suppressed by licking (L+, L−) or forelimb pulling (F+, F−). Neurons responsive to both movements showed either a mutual (L+/F+, L−/F−) or opposite modulation (L+/F−, L−/F+) during each motor task. We found that PNs (Fig. 9*B*, violet bars) were distributed across all functional classes. In contrast, the vast majority of FSNs (>72%) were mutually modulated (i.e., suppressed or enhanced) by the two different movements (i.e., L+/F+, 50% and L−/F−, 20%) showing a broader tuning than PNs (Fig. 9*B*), similarly to the data previously reported for “single” and “multiple” licks (Fig. 3*G*). However, licking was the preferred neuronal response for all recorded units, and even considering those neurons activated by both movements, the average peak firing rate (Fig. 9*C*) and the intensity of activation (Fig. 9*D*) were significantly lower during forelimb than during licking activity both in FSNs and PNs, consistently with the anatomic location of the implanted array. To assess whether PNs and FSNs licking/forelimb preference was related to the location inside ALM, we compared the proportion of all forelimb-related neurons among electrode positions, over the region covered by the 4 × 4 chronic microelectrode array in the implanted mice (see Materials and Methods). Overall, we found no evidence for a clear segregation of function at the level of single neurons in the ALM and the portion of sampled RFA, neither for PNs nor for FSNs (one-way ANOVA: PNs, *F*_(15,23)_ = 0.97, *p* = 0.51; FSNs, *F*_(11,5)_ = 1.26, *p* = 0.42; Fig. 9*E*,*F*).

**Figure 10.**
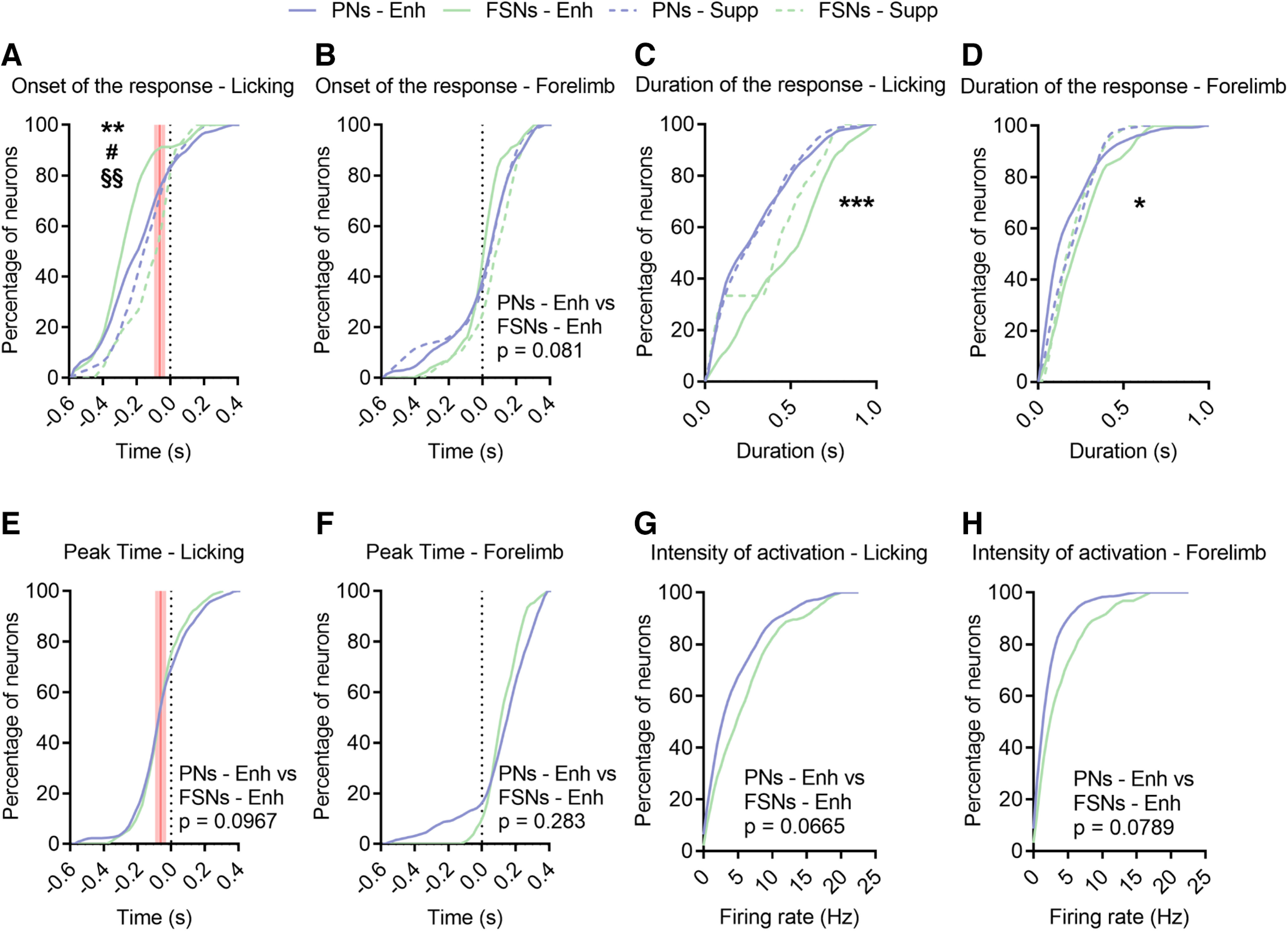
FSNs show more sustained activation than PNs during forelimb pulling. ***A***, Cumulative distribution of the onset of the response for all neurons during a licking bout. The red shaded lines indicate the confidence interval (61.9 ± 20.8 ms) of movement initiation, the first mouth movement before licksensor activation. Enhanced neurons are represented as continuous lines (PNs, violet; FSN, green); dotted lines indicate the suppressed PNs and FSNs. Enhanced PNs versus suppressed PNs, K–S test, #*p* = 0.043. Enhanced FSNs versus suppressed FSNs, K–S test, §§*p* = 0.0090. Enhanced PNs versus enhanced FSNs, K–S test, **p = 0.0069. ***B***, Cumulative distribution of the onset of the response (*t* = 0 corresponds to force peak beginning) for all neurons during the forelimb retraction. Enhanced PNs versus suppressed PNs, K–S test, *p* = 0.91. Enhanced FSNs versus suppressed FSNs, K–S test, *p* = 0.12. Enhanced PNs versus enhanced FSNs, K–S test, *p* = 0.081. ***C***, Cumulative distribution of the duration of the response for all neurons during a licking bout. Enhanced FSNs versus suppressed FSNs, K–S test, *p* = 0.610. Enhanced PNs versus suppressed PNs, K–S test, *p* = 0.987. Enhanced PNs versus enhanced FSNs, K–S test, ***p = 0.0009. ***D***, Cumulative distribution of the duration of the response for all neurons during the forelimb retraction. Enhanced PNs versus suppressed PNs, K–S test, *p* = 0.137. Enhanced FSNs versus suppressed FSNs, K–S test, *p* = 0.216. Enhanced PNs versus enhanced FSNs, K–S test, *p = 0.029. ***E***, Cumulative distribution of the peak time for all neurons during a licking bout. Enhanced PNs versus enhanced FSNs, K–S test, *p* = 0.0967. Red shaded lines, as in ***A***. ***F***, Cumulative distribution of the peak time for all neurons during the forelimb retraction. Enhanced PNs versus enhanced FSNs, K–S test, *p* = 0.283. ***G***, Cumulative distribution of the intensity of activation for all neurons during a licking bout. Enhanced PNs versus enhanced FSNs, K–S test, *p* = 0.0665. ***H***, Cumulative distribution of the intensity of activation for all neurons during the forelimb retraction. Enhanced PNs versus enhanced FSNs, K–S test, *p* = 0.0789.

We next compared the response onset and duration among the different populations of neurons. Consistently with results in acute recordings, concerning licking activity (Fig. 10*A*) enhanced FSNs started to discharge before facilitated PNs. Instead, during forelimb pulling a significant earlier activation of FSNs was not confirmed (Fig. 10*B*) since, as in laminar recordings (Fig. 7), a subset of pyramidal neurons (∼15%) modulated their discharges very early. Interestingly, the suppressed FSNs showed a delayed discharge onset relative to the enhanced FSNs, especially during licking (Fig. 10*A*,*B*).

In terms of duration of the response, this was significantly greater for the FSNs, specifically those excited during movement, considering both licking (Fig. 10*C*) and pulling (Fig. 10*D*). The suppressed FSNs showed a shorter duration of modulation, although not statistically different from that of enhanced FSNs (Fig. 10*C*,*D*). There was no difference in the discharge duration between enhanced and suppressed PNs (Fig. 10*C*,*D*).

The peak time was not modulated in enhanced FSNs compared with enhanced PNs during both licking (Fig. 10*E*) and forelimb retraction (Fig. 10*F*) while a general trend toward a greater intensity of activation was found in enhanced FSNs with respect to PNs during both types of movements (Fig. 10*G*,*H*).

Altogether, these data support the previous laminar recordings in indicating an early and prolonged discharge of FSNs activated by licking, but not pulling, suggesting a specificity of the early inhibitory tone for the primary body effector associated to the considered area. Interestingly, the suppressed FSNs were modulated at longer latencies during movement generation.

### Causal role of FSNs activation in movement facilitation

**Figure 11.**
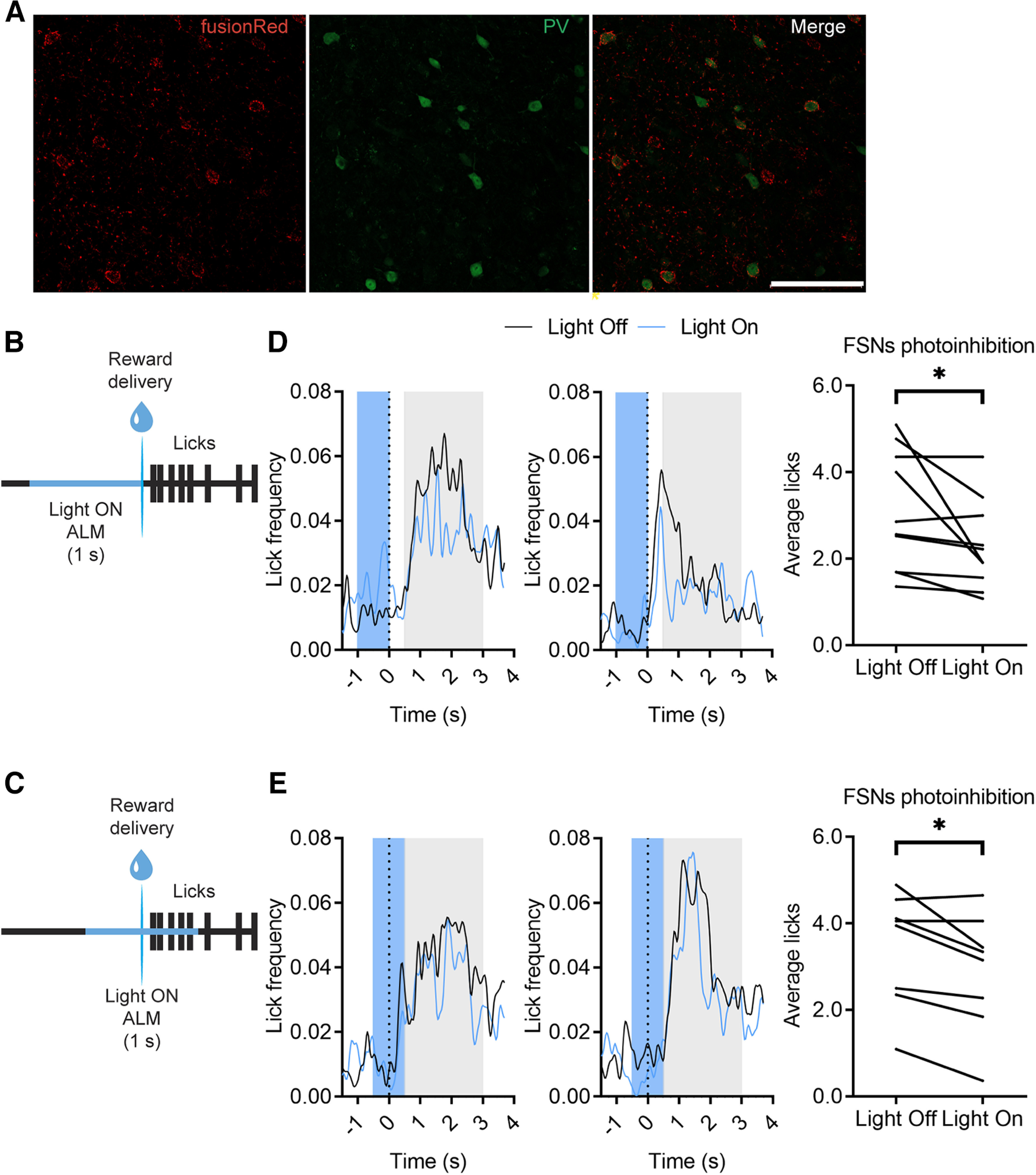
Optogenetic FSNs inhibition reduced licking behavior. ***A***, Representative ALM micrograph (20×) of a PV-Cre mouse injected with the floxed AAV5-stGtACR1-FusionRed. FusionRed reporter (red) shows specific expression of the floxed AAV in Parvalbumin-positive (PV) neurons (green), stained with immunohistochemistry. Scale bar, 100 µm. ***B***, ***C***, Schematic of licking tasks with optogenetic silencing of FSNs in the right ALM for 1 s. The inhibition starts 1 s (***B***) or 0.5 s (***C***) before the reward delivery onset and lasts until the liquid drop delivery or 0.5 s later, respectively. ***D***, ***E***, Frequency distribution of licks during the licking task of 2 mice during the Light Off (black traces) and the Light On (blue traces) trials. The dotted black lines (0 s) indicate the reward delivery. Blue shaded areas represent the ALM PV+ FSNs optogenetic inhibition interval in Light On trials. Graphs on the right represent average licks for each session (*n* = 4–5) of the two mice in a 2.5-s interval (gray shaded areas of the left graphs), during the light off and light on trials. Top, Paired *t* test, *t*_(1,9)_ = 2.30, *p = 0.0468. Bottom, Paired *t* test, *t*_(1,7)_ = 3.068, *p = 0.018.

To assess a causal role of FSNs activity in licking movements we inhibited PV+ FSNs expressing anion-conducting ChR2 in the right ALM of PV-Cre mice (Fig. 11*A*). In a first set of mice (*n* = 2), trials with 1 s of optogenetic silencing of FSNs before reward delivery were pseudorandomly administered together with an equal number of trials with no stimulation (Fig. 11*B*). In a second set of mice (*n* = 2) the 1 s optogenetic inhibition was started 0.5 s before reward delivery onset (Fig. 11*C*). We found that licking activity, monitored through the lick sensor after reward delivery, was significantly reduced in both cases during optogenetic inhibition (Fig. 11*D*,*E*, blue traces) compared with control trials (Fig. 11*D*,*E*, black traces). These data suggest that inhibitory neurons in the mouth region have a causal role in facilitating spontaneous licking movement.

## Discussion

In the present study, we demonstrated that FSNs in the mouth/face motor field of the mice anterolateral premotor cortex fire in anticipation of PNs with a specific pattern of activation during spontaneous licking, but not during forelimb movements. FSNs become active earlier, longer, and more intensely than PNs, also carry more information about movement onset than PNs. Furthermore, this rise of inhibitory activity appears to causally contribute to the initiation and execution of actions, as suggested by the results of our optogenetic silencing experiments. These findings are in agreement with a previous electrophysiological study examining the discharge of FSNs and PNs in mouse primary motor cortex during sensory-triggered as well as voluntary forelimb reaching movements (Estebanez et al., 2017), and support a role of early inhibition mediated by FSNs during motor activity by both primary motor and premotor areas.

Preparatory/ramping activity in ALM PNs has been shown to be maintained by a recurrent excitatory loop that involves both the cortex and the ipsilateral thalamus (Guo et al., 2017). Since FSNs are directly reached by thalamic afferents (Lourenço et al., 2020b), this recurrent thalamocortical loop may sustain persistent firing activity observed in FSNs. It is worth noting that, although PNs were recruited later than FSNs during movement initiation in our study, a fraction of PNs located in deep layers, was early-modulated. Despite the sampling bias of laminar recordings, which clearly favors the sampling of deep relative to superficial neurons and hence suggest cautiousness in interpreting these findings, we reported a generally earlier involvement of deeper neurons during licking behaviors. In particular, early-modulated deep PNs may represent preparatory “master” neurons that subsequently command downstream, more executive PNs and FSNs. In keeping with our results, which concerns spontaneous behavior, it has been shown that preparatory activity appears first in deep layers of ALM during a task with an instructed, delayed motor response (Chen et al., 2017). Concerning FSNs suppressed during movement execution, the onset data clearly show that they are consistently delayed with respect to the other populations. Since PV+ FSNs form a highly interconnected set of neurons (Lourenço et al., 2020a), it is likely that the suppressed fast-spiking population receives direct synaptic input from enhanced FSNs.

A general finding that applies to all types of recorded neurons is that the great majority of them do not fire in relation to individual licking movements, nor are influenced by the number of licking movements in a bout (i.e., multiple vs single licks). Nonetheless, FSNs were less selective for the movement type than PNs, which in turn exhibited a richer variety of behaviors, from enhanced to suppressed discharge depending on the specific movement in relation to which they fired (i.e., licking vs pulling). In contrast, the percentage of suppressed FSNs was lower, and they often increased their firing rate during both pulling and licking movement, thus showing lower motor specificity. Accordingly, FSNs appear to carry more redundant information than PNs, consistently with the fact that FSNs are known to be synchronized by electrical and chemical synapses (Lourenço et al., 2020b). In fact, previous studies showed that in the prefrontal cortex of mice performing a sensory discrimination task, PV+ FSNs were activated by all movement-related events (sensory cues, motor action, and trial outcomes), while responses of PNs were diverse and more selective (Pinto and Dan, 2015). The broader tuning of FSNs is also consistent with previous findings in sensory cortices, where interneurons were poorly selective for stimulus features such as orientation (Kerlin et al., 2010; Hofer et al., 2011), and in monkey parieto-premotor cortices, as shown by recent evidence concerning visual and motor tuning for object type during visually-guided grasping actions (Ferroni et al., 2021).

An additional important finding is that we could not identify a clear tuning map for the two investigated movements (licking and forelimb retraction), which involve two distinct effectors, neither when PNs nor when FSNs were considered. From a comparative point of view, these results are consistent with the findings in the monkey ventral premotor cortex, in which forelimb and face/mouth representations largely overlap both in terms of functional properties and electrically-evoked motor responses (Maranesi et al., 2012). Coherently, intracortical microstimulation (ICMS) of the frontal cortex in mice showed a highly variable distribution of sites leading to forelimb/head movements in individual animals (Tennant et al., 2011), suggesting that anatomic overlapping between the cortical representation of functionally-related effectors is an evolutionarily conserved solution for motor control.

It has been hypothesized that the activity of interneurons, including FSNs, provides an inhibitory gate that prevents preparatory activity from causing undesired movements. If this were the case, interneuron firing rates should be reduced around the time of movement, which was not observed in the present experiments. Another possibility is that FSN-mediated inhibition may serve to suppress other actions (e.g., movement of other body parts). If FSNs act to prevent adjacent cortical modules from producing other movements, one would predict the existence of distinct licking-related and forelimb-related FSNs which reciprocally inhibit the respective PNs. However, our data do not provide support for such a model, as >50% of FSNs increase their discharge during both licking and forelimb retraction. Thus, a sustained, overall rise in FSNs activity appears to be required, likely to reach a critical level of inhibition for properly releasing and maintaining motor activity. To probe this hypothesis, we employed optogenetic silencing of FSNs activity prior and during reward delivery, demonstrating that in both cases there was a significant reduction in the frequency of spontaneous licking behavior during the time period following the stimulation, supporting the idea that FSNs activity play a role in the initiation and maintenance of sequential motor actions.

Despite the increase of inhibitory activity is known to be a general phenomenon linked to movement execution, anticipation of FSNs activity across all cortical layers appears to be specific for the motor action primarily represented in the investigated motor field. In fact, we focused our study on the ALM, which is an area primarily involved in the control of licking and mouth-related actions: coherently, we observed a prevalence of neurons (both PNs and FSNs) responding to licking rather than forelimb actions, and virtually no FSNs selectively activated during forelimb movements. While in our study on a mouth/face premotor region we found early FSNs activity during licking but not during forelimb retraction, early FSNs activation has been reported during forelimb movements when recordings were conducted in the forelimb motor cortex (Estebanez et al., 2017), supporting a specific role of FSNs in shaping and sculpting the motor output primarily influenced by a given cortical sector, likely acting on the response selectivity of nearby PNs.

In the motor cortex, the magnitude of inhibition directly affects tuning of individual PNs before and during movement execution both in mice (Galiñanes et al., 2018) and nonhuman primates (Georgopoulos et al., 1982; Merchant et al., 2008). Furthermore, the activity of FSNs might provide an inhibitory constraint that maintains firing rates of PNs within an “optimal subspace” (Afshar et al., 2011) that allows accurate movement (Churchland et al., 2006). Future studies should address these alternative hypotheses on the mechanistic role of FSNs in contributing to specification and initiation of voluntary movements.

In conclusion, our study contributes to clarifying the causal role of FSNs in driving, with a global rise of inhibition, the initiation and execution of specific, spontaneous motor actions by mouse premotor cortex.
